# All-flesh fruit in tomato is controlled by reduced expression dosage of *AFF* through a structural variant mutation in the promoter

**DOI:** 10.1093/jxb/erab401

**Published:** 2021-09-07

**Authors:** Lei Liu, Kang Zhang, Jinrui Bai, Jinghua Lu, Xiaoxiao Lu, Junling Hu, Chunyang Pan, Shumin He, Jiale Yuan, Yiyue Zhang, Min Zhang, Yanmei Guo, Xiaoxuan Wang, Zejun Huang, Yongchen Du, Feng Cheng, Junming Li

**Affiliations:** 1 Key Laboratory of Biology and Genetic Improvement of Horticultural Crops of Ministry of Agriculture, Institute of Vegetables and Flowers, Chinese Academy of Agricultural Sciences, Beijing 100081, China; 2 University of Trento, Italy

**Keywords:** All-flesh fruit, *cis*-regulatory mutation, dosage effect, locule gel, processing tomato, *Solanum lycopersicum*, structural variant

## Abstract

The formation of locule gel is an important process in tomato and is a typical characteristic of berry fruit. In this study, we examined a natural tomato mutant that produces all-flesh fruit (AFF) in which the locule tissue remains in a solid state during fruit development. We constructed different genetic populations to fine-map the causal gene for this trait and identified *SlMBP3* as the locus conferring the locule gel formation, which we rename as *AFF*. We determined the causal mutation as a 416-bp deletion in the promoter region of *AFF*, which reduces its expression dosage. Generally, this sequence is highly conserved among Solanaceae, as well as within the tomato germplasm. Using BC_6_ near-isogenic lines, we determined that the reduced expression dosage of *AFF* did not affect the normal development of seeds, whilst producing unique, non-liquefied locule tissue that was distinct from that of normal tomatoes in terms of metabolic components. Combined analysis using mRNA-seq and metabolomics indicated the importance of *AFF* in locule tissue liquefaction. Our findings provide insights into fruit-type differentiation in Solanaceae crops and also present the basis for future applications of *AFF* in tomato breeding programs.

## Teaser

All-flesh fruit in tomato is controlled by a sequence deletion in the *cis*-regulatory region of the promoter of *AFF* that results in reduced expression dosage and the production of non-liquefied locule tissue.

## Introduction

Locule gel is a typical characteristic of berry fruit. One important example is tomato (*Solanum lycopersicum*), which is used as a model plant for the study of fruit development and ripening as its fruit has clear tissue distribution and structure (Huber and Lee, 1986; Joubès *et al.*, 1999; Czerednik *et al.*, 2015). A large body of literature on tomato fruit development exists, but is mainly focused on the type, weight, and ripening of the fruit. A number of studies have comprehensively described the molecular activity within developing locules through large-scale transcriptional analyses together with physiological and biochemical surveys (Lemaire-Chamley *et al.*, 2005; Seymour *et al.*, 2008; Lin *et al.*, 2014; Azzi *et al.*, 2015; Shinozaki *et al.*, 2018; Zhu *et al.*, 2018). However, the regulation of locule gel formation and development remains unclear.

Tomato locule tissue is the second-most abundant tissue in tomato fruit and represents 23% (w/w) of the fresh weight (Mounet *et al.*, 2009). The formation of locule tissue has been shown to be a complex process involving a series of physiological and biochemical changes that play critical roles in fruit growth and maturation (Lemaire-Chamley *et al.*, 2005; Mounet *et al.*, 2009; Azzi *et al.*, 2015). Tomato locule tissue generally derives from the placenta and grows up around the ovules (Davies and Cocking, 1965). It encloses the developing seeds, undergoes extensive expansion and liquefaction, and transforms into a jelly-like, homogenous tissue that is composed of giant, thin-walled cells (Atherton and Rudich, 1986; Cheng and Huber, 1996; Joubès *et al.*, 1999). However, the regulation of locule differentiation and formation during the development of the fruit remains unknown. The natural tomato mutant ‘all-flesh fruit’ (named here as AFF) does not produce locule gel, and this completely alters the structure of the locule tissue (Silvestri, 2006; Macua *et al.*, 2015). It therefore potentially provides ideal material for examining the regulation mechanism behind locule development. This mutant also offers several advantages for the tomato-processing industry, such as its high content of solids, improved firmness, long shelf-life, and color characteristics compared to wild-type tomato (Silvestri, 2006; Macua *et al.*, 2015). Detailed examination of AFF might therefore be beneficial not only for improving our knowledge of berry fruit formation, but also for breeding programs.

Phytohormones and cell wall-modifying enzymes have commonly been considered to play important roles in locule gel formation. Evidence clearly indicates that IAA, GA, and ABA are present at high levels in seeds and are transported to the surrounding tissues where they participate in inducing and regulating the development of locule tissue (Lemaire-Chamley *et al.*, 2005; Moco *et al.*, 2007; Mounet *et al.*, 2009; Kumar and Khurana, 2014). However, it has been demonstrated that ethylene and IAA do not control the determination and liquefaction of locule gel in tomato fruit (Brecht, 1987; Gillaspy *et al.*, 1993; Qin *et al.*, 2012). Instead, the formation of locule gel might be related to the ripening and softening of fruit, because development of the gel progresses alongside the dissolution of pectin, deglycosylation, and hemicellulose—the main components of the cell wall matrix—as catalysed by polygalacturonase (PG) and pectin methylesterase (PME) (Cheng and Huber, 1997; Nunan *et al.*, 1998; Bapat *et al.*, 2010). However, PG and PME mainly change the texture of fruit and do not determine the process of locule gel formation (Tieman *et al.*, 1992; Uluisik *et al.*, 2016). The initial formation of the gel might therefore involve a mechanism that is different from the classic phytohormones and/or PME–d-galacturonanase scenarios.

The well-known floral ‘ABCDE’ model was developed to describe the regulation of floral organ development and differentiation. The D-class genes contribute to the formation of the seeds, ovule, and funiculus and regulate the expansion and maturation of the carpel and fruit (Vrebalov *et al.*, 2009; Itkin *et al.*, 2010; Dreni and Kater, 2014). For example, the first set of D-class MADS-box genes reported in petunia, *FLORAL BINDING PROTEIN 7* (*FBP7*) and *FBP11*, are expressed specifically in ovule differentiation and also participate in seed and coat development (Angenent *et al.*, 1995; Colombo *et al.*, 1995). Another orthologous gene, *SEEDSTICK* (*STK*; previously *AGL11*), isolated from Arabidopsis, is also involved in the development of ovules and affects seed germination (Favaro *et al.*, 2003; Pinyopich *et al.*, 2003; Ezquer *et al.*, 2016). Suppression of *STK* orthologs in tomato and grape triggers seedless fruits (Ocarez and Mejía, 2016), whereas overexpression of tomato *TAGL11* results in dramatic modifications of flower and fruit organization (Huang *et al.*, 2017). In addition, *SHATTERPROOF1* (*SHP1*) and *SHP2* act redundantly with *STK* in promoting ovule identity (Liljegren *et al.*, 2000; Pinyopich *et al.*, 2003). Similarly, in tomato, *TAGL1*, an ortholog of *SHP*, controls fruit expansion and fleshiness (Vrebalov *et al.*, 2009). More recently, Zhang *et al.* (2019) found that the D-class gene AGAMOUS MADS-box protein 3 (*SlMBP3*)—a paralog of *TAGL1*—modulates both placenta liquefaction and seed formation in tomato,with RNAi and overexpression lines of *SlMBP3* yielding fleshy fruit without locular gel and with accelerated placenta liquefaction, respectively. Furthermore, the *SlMBP3*-RNAi lines produced malformed seeds that were not able to germinate, while the overexpression lines generated larger seeds. In addition, it is a common feature that all these D-class genes participate in seed development, such as Arabidopsis *STK*, the mutant of which exhibits reduced seed germination efficiency (Ezquer *et al.*, 2016), tomato *TAGL11*, which maintains the production of seeds (Huang *et al.*, 2017), and tomato *SlMBP3*, RNAi plants of which produce seeds that are not able to germinate (Zhang *et al.*, 2019). In contrast, the natural AFF mutant described above produces normal seeds with a high germination rate, and it therefore provides a novel system for exploring the genetic mechanisms underlying ovule development, and especially locule gel formation, without the presence of negative effects on seed development.

In this study, we fine-mapped the *AFF* gene by combining a genetic analysis and map-based cloning approach. We found that a novel structural variant, a 416-bp sequence deletion, occurred in the conserved *cis*-regulatory region of *aff*. This deletion suppressed the expression of *AFF* and produced the all-flesh fruit phenotype. In addition, we performed combined transcriptome and metabolome analyses using *aff* near-isogenic lines to examine the regulatory pathways and the effects on fruit quality of the mutation, and found that the metabolic components showed distinct differences compared with the wild-type. Our findings provide novel insights into the evolution of berry fruits together with useful information for tomato breeding programs.

## Materials and methods

### Plant materials and crossing

Tomato (*Solanum lycopersicum*) plants were cultivated under greenhouse conditions at the Institute of Vegetables and Flowers, Chinese Academy of Agricultural Sciences (IVF-CAAS), Beijing, China, during the natural growing season. Seeds of the lines 06-790 and 09-1225 (all-flesh fruit, *aff*) were obtained from our own stocks (China National Vegetable Germplasm Bank at IVF-CAAS). Seeds of the lines LA4069, H1706 (which was used for the tomato genome sequencing project; Tomato Genome Consortium, 2012), and Micro Tom were obtained from the Tomato Genetics Resource Center (TGRC) at the University of California, Davis, USA.

The *aff* line 06-790 was crossed with the wild-type (WT) LA4069 to generate F_1_ progeny, and F_2_ progeny were derived from self-pollination of them. The F_1_ progeny were back-crossed with 06-790 to generate BC_1_P1 progeny and back-crossed with LA4069 to generate BC_1_P2 progeny. All six populations of two crosses were grown for genetic analysis in the greenhouse in the spring of 2015.

BC_2_S_1_ progeny were developed from the *aff* line 06-790 as donor parents, with continued backcrossing to H1706. Two individuals showing the *aff* phenotype (BA-130 and BA-150) and two individuals showing the normal phenotype (BA-124 and BA-128) were then selected for qRT-PCR analysis.

Near-isogenic lines (NILs) of *aff* were derived from BC_6_S_2_ plants generated by continual backcrossing of 06-790 to H1706. We selected two NILs showing the *aff* phenotype (BA-1 and BA-2) and two individuals showing the normal phenotype (BA-4 and BA-6) for assessment of seed germination. BA-1 and H1706 were also used for examination of morphology research and for transcriptome and metabolome profiling.

### Paraffin sectioning

Fruits were sampled from plants at 25 d after flowering (DAF). Locule tissue was cut into 1×2×2 mm cubes and fixed in FAA (5% acetic acid, 5% formaldehyde, 50% ethanol, 5% glycerin mixture) for 24 h at room temperature. After pre-treatment of dehydration in an alcohol series, embedding in paraffin, slicing with an ultra-thin semi-automatic microtome, and dewaxing in xylene, sections were dyed using Safranin O and Fast Green double-dye, according to Wang and He (2004). The paraffin sections were examined and imaged using an Olympus IX71 microscope.

### Genome sequencing, SNP and SV calling, and KASP analysis

For rapid identification of the mutation conferring the all-flesh fruit phenotype in 06-790 we used MutMap, a method based on whole-genome resequencing of bulked DNA of F_2_ segregants (Takagi *et al.*, 2013). We designed two mixed-DNA pools that combined 30 F_2_ progeny that had either the AFF phenotype or the normal phenotype. These pools were subjected to whole-genome resequencing using an Illumina GAIIx DNA sequencer (Beijing Berry Genomics Co., Ltd). The sequencing depth was ~20-fold coverage for the two parental lines and ~30-fold coverage for the two mixed-DNA pools. The paired-end reads of 06-790, LA4069, and the mixed-DNA pools were mapped to the tomato reference genome (SL4.0 build; ITAG4.0 annotation; Tomato Genome Consortium, 2012) using Burrows–Wheeler Aligner (version 0.7.10-r789) with default parameters (Li and Durbin, 2009). The BAM files were further deduplicated using the MarkDuplicate function of Picard (http://broadinstitute.github.io/picard/). The HaplotypeCaller function of GATK was used to call the variants with the default parameters (Mckenna *et al.*, 2010). Variants supported by less than three reads were filtered out. ANNOVAR was used to annotate the retained variants (Wang *et al.*, 2010). The ∆SNP-index was calculated based on a 200-kb sliding window with a 20-kb increment. The potential structural variants (SVs) of the *aff* line were called using BreakDancer (Version 1.1.2, http://gmt.genome.wustl.edu/breakdancer/current/) based on the BAM file. A total of 24 single-nucleotide polymorphisms (SNPs) with strong associations were selected to develop kompetitive allele specific PCR (KASP) markers, and the SNP analysis and genotyping of populations were conducted using the KASP genotyping system (LGC Genomics). Among these SNPs, 13 that differed between the AFF and normal plants were used for fine-mapping (Supplementary Table S1).

### Conservation of the *AFF* promoter sequence between orthologous genes

Syntenic orthologous genes of *AFF* among Solanaceae crop species were determined using the SynOrths tool (Cheng *et al.*, 2012), namely *S. lycopersicum* (*Solyc06g064840*), *S. pennellii* (*Sopen06g023350*), *S. tuberosum* (*Sotub06g020180*), *S. melongena* (*Sme2.5_02049.1_g00007.1*), and *Capsicum annuum* (*Capang01g002169*). We then extracted 5-kb upstream sequences (promoter region) of each of the five orthologous genes from the genomes of the five species. These sequences were further aligned using MUSCLE (Edgar, 2004). The aligned sequences were used to calculate the conservation level of each aligned nucleotide and then averaged with a 50-bp sliding window with a step of 10 bp using an in-house Perl script (available upon request).

We also investigated the sequence conservation of the *AFF* gene in the tomato germplasm using the published variome datasets of 360 tomato samples (Lin *et al.*, 2014). We calculated the nucleotide diversity (π) values for the 3-kb upstream region, gene body, and 3-kb downstream region for all 34 075 tomato genes in the genome of *S. lycopersicum* with the variome datasets using VCFtools (Danecek *et al.*, 2011). The distributions of the π values in the three regions were plotted as bean-plots using the R package ‘beanplot’ (Kampstra, 2008).

### RNA extraction and qRT-PCR

The different tomato lines were detected by quantitative real-time PCR with the use of specific primers and probes. They included the *aff* lines BA-130, BA-150, 06-790, and 09-1225, the WT lines BA-124, BA-128, LA4069, and H1706, and the F_1_ progeny from the crossing of 06-790 and H1706. Plants were grown in the greenhouse in the autumn of 2017, and RNA was collected from locule tissues at 7, 10, 15, and 25 DAF, with three samples taken per line. *SlFRG27* (*Solyc06g007510*), *SlFRG03* (*Solyc02g063070*), and *ACTIN* (*Solyc11g005330*) were selected as the reference genes, and the primer sequences are listed in Supplementary Table S2 (Cheng *et al.*, 2017). The primer sequence of *AFF* was F (5´–3´), GCATCTGGTTGGTGAAGG; R (5´–3´), ATCTGATTCTGCTGATGCC. The primers were designed using the Roche LCPDS2 software and synthesized by Beijing TsingKe Biological Technology Co., Ltd. cDNA was obtained from total RNA by reverse-transcription using a PrimeScript RT reagent kit (TaKaRa). The qRT-PCR was conducted on a Prism^®^7900 qRT-PCR operating system (Applied Biosystems), according to the instructions of the SYBR Prime Script RT-PCR kit. The 2^–ΔΔ*C*T^ method was used to determine the expression of the genes.

### Gene knock-out and overexpression

To knock-out *AFF*, two sgRNAs targeting the second and the third exon of the gene were designed and constructed into the CRIPSPR/Cas9 expression vector BGK012-DSG to obtain the recombinant plasmids MSG8124/8125. The plasmids were introduced into cotyledon explants of *S. lycopersicum* cv. Micro Tom (WT) through *Agrobacterium tumefacien*-mediated transformation, as described previously (Sun *et al.*, 2006). The transgenic plants were confirmed by genotyping PCR using Sanger sequencing. For overexpression of *AFF* (*Solyc06g064840*), the full-length coding sequence (CDS) was cloned into vector pEXT06/g to construct the recombinant plasmid *35S::AFF-CDS::GFP*. The plasmid was then introduced into Micro Tom to obtain transgenic plants with overexpression.

### Dual-luciferase assays

Having identified a 416-bp deletion in the promoter region of *AFF*, we conducted dual luciferase reporter assays to confirm its function in modifying expression. Using a PCR-based accurate synthesis method, full-length splicing primers were designed and the protective base synthesis gene promoters (Del and WT) at both ends of the primers were inserted into sites between PvuII and KpnI in the plasmid pGreenII 0800-LUC. The recombinant plasmid pGreenII 0800-LUC-promoter(Del) was transferred into the epi400 clone strain, and the recombinant plasmid pGreenII 0800-LUC-promoter(WT) was transferred to the Top10 clone strain. The sequence of the recombinant plasmid was verified by the sequence of the positive clones.

Monoclones were selected for PCR verification after plasmid transformation. Leaves of 1-month-old *Nicotiana benthamiana* plants were transiently infected by positive strains using an *Agrobacterium*-mediated method. Each group had three replicates. The activity of the dual luciferase reporter gene was detected after 3 d, and the transcriptional regulation was determined by the activity ratio of firefly luciferase and *Renilla* luciferase (LUC/REN ratio).

### Metabolic network analysis using transcriptome and metabolome profiling

Metabolome profiling was carried out using a widely targeted metabolome method by Metware Biotechnology Co., Ltd (Wuhan, China; http://www.metware.cn/). Samples of whole fruits and locule and placenta tissues were taken from plants at 10, 15, and 25 DAF. Briefly, the tomato tissues were lyophilized and ground into fine powder using a mixer mill (MM 400, Retsch) with zirconia beads for 1.5 min at 30 Hz. Then, 100 mg of the powder was weighed and extracted overnight with 1.0 ml 70% aqueous methanol at 4 °C, followed by centrifugation for 10 min at 10 000 *g*. All supernatants were collected and filtered through a membrane (SCAA-104, 0.22 mm pore size; ANPEL, Shanghai, China; http://www.anpel.com.cn/) before LC-MS analysis. Quantification of metabolites was carried out using a scheduled multiple reaction monitoring method (Wei *et al.*, 2013; Zhu *et al.*, 2018). In the data analysis process, unsupervised principal component analysis (PCA) was performed using the function prcomp within R (version 3.5.0; www.r-project.org). The data were unit-variance scaled before performing the unsupervised PCA. The results for hierarchical cluster analysis (HCA) of samples and metabolites are presented as heatmaps with dendrograms, while Pearson correlation coefficients between samples were calculated using the cor function in R. Both HCA and calculation of Pearson correlations were carried out using the R package pheatmap (version 1.0.12). Identified metabolites were annotated using the Kyoto Encyclopedia of Genes and Genomes (KEGG) COMPOUND database (http://www.kegg.jp/kegg/compound/) and then mapped to the KEGG pathway database (http://www.kegg.jp/kegg/pathway.html). Pathways with significantly regulated metabolites were then fed into MSEA (Xia and Wishart, 2010).

For RNA-sequencing, which was conducted on the same tissue samples as for the metabolite profiling, a total of 3 µg RNA per sample was used as input material. Sequencing libraries were generated using a NEBNext^®^ Ultra™ RNA Library Prep Kit for Illumina^®^ (New England Biolabs) following the manufacturer’s instructions, and index codes were added to attribute sequences to each sample. The constructed libraries were then sequenced on an Illumina HiSeq platform, and 125/150-bp paired-end reads were generated. Transcriptome profiling was performed as described previously (Ying *et al.*, 2020). Briefly, clean reads were obtained using a HiSeq-X-ten sequencing platform, mapped to the tomato reference genome (version 4.0) using HISAT2 (Kim *et al.*, 2015), and then normalized to TPM (tags per million) reads using StringTie (Pertea *et al.*, 2015).

Samples of locule and placenta tissues that were taken from plants at 10, 15, and 25 DAF were analysed using MeV (version 4.9) with the *k*-means method (Gasch and Eisen, 2002). HCA and PCA were performed to determine relatedness among the different time-points/tissue samples.*Z*-scores derived from the transformed and normalized gene and metabolite expression values were used for HCA and PCA. We used Pearson’s correlation algorithm method (Bishara and Hittner, 2012) to construct a transcription factor-related gene and metabolite regulatory network. Mutual information was used to calculate the expression similarity between the expression levels of transcription factors and genes, and metabolite pairs were calculated using the R software. All the associations among transcription factors, genes, and metabolites were analysed using the Cytoscape software (Kohl *et al.*, 2011).

### Phylogenetic analysis

Protein sequences of genes that were homologous to *AFF* were collected from the NCBI database through the BLAST service. These protein sequences were then aligned using Clustal Omega (Madeira *et al.*, 2019), and the multiple alignments were then used to construct a phylogenetic tree using IQ-TREE with the maximum likelihood method (Nguyen *et al.*, 2015). Protein structure analysis was performed with the assistance of the Pfam server (Finn *et al.*, 2008).

## Results

### The all-flesh fruit trait is controlled by a single recessive locus

To investigate the genetic characteristics of the tomato *aff* genotype, we constructed an F_2_ population using the *aff* genotype 06-790 as parent P1 and the WT LA4069 as parent P2. Examination of the fruit traits of the F_2_ population showed that the ratio of WT to *aff* samples was 150:41, consistent with a 3:1 segregation (chi-squared test: *χ*^2^=1.176, *P*>0.05; Table 1), suggesting a single recessive genetic model of the AFF trait. To further confirm this, we constructed a BC_1_P1 population. The progenies showed a ratio of WT to *aff* samples of 46:42, conforming to a 1:1 segregation ratio (*χ*^2^=0.102, *P*>0.05). We also constructed a BC_1_P2 population, and all progenies of this population had WT fruit. Taken together, the data confirmed that the AFF trait is controlled by a single recessive mutation.

**Table 1.** Segregation of the normal *versus* all-flesh fruit trait in populations derived from the *aff* parent (P1) and the wild-type parent (P2)

Generation	Population size	Segregation		Theoretical ratio	χ^2^	Significance
		Normal	All-Flesh			
P1 (06-790)	20	0	20	–	–	–
P2 (LA4069)	20	20	0	–	–	–
F_1_	20	20	0	–	–	–
F_2_	191	150	41	3:1	1.176	*P*>0.05
BC_1_P1	88	46	42	1:1	0.102	*P*>0.05
BC_1_P2	40	40	0	–	–	–

### Locule cells in *aff* fruit maintain their structure during ripening

**Fig. 1.**
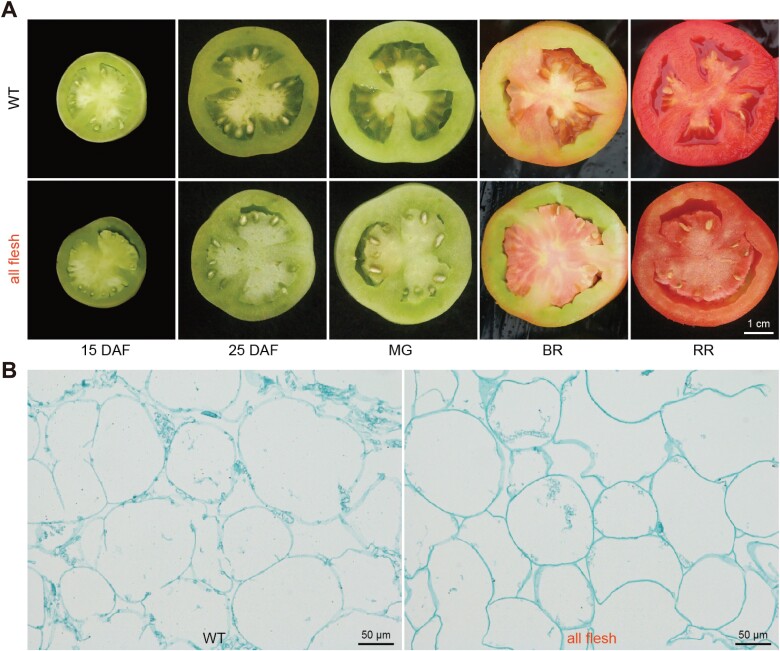
Morphology of the locule tissues in wild-type and all-flesh fruit tomato. (A) Representative images of the locule tissue at different developmental stages of the wild-type (WT) line LA4069 and the *aff* line 06-790. DAF, days after flowering; MG, mature green; BR, breaker ripe; RR, red ripe. (B) The cell structure of locule tissues of the WT and *aff* lines at the mature green stage.

To determine the time-point for the initiation of locule gel development, we first observed the difference in locule tissue between *aff* and WT plants by cutting fruits at 5-d intervals. We found that no jelly-like tissue formed in the locule cavity area during the whole development process of the *aff* genotype (Fig. 1A), whereas in contrast obvious jelly-like tissue was observed in the WT at 25 DAF, and the fruit reached complete liquefaction after the mature green (MG) stage (~30 DAF). This indicated that 25 DAF was an important time-point for the formation and development of the locule gel. We therefore examined the microscopic structure of the locule tissues in paraffin sections of fruit at the MG stage, and found that individual cells of the WT were collapsing and showed a tendency to fracture inter-cellularly within the plane of the cell wall (Fig. 1B), which was consistent with previous reports (Cheng and Huber, 1996; Lemaire-Chamley *et al.*, 2005). In contrast, these distinct changes did not occur in cells of the *aff* locule, which instead maintained their structure. The morphology of the locule tissue in the *aff* fruit was thus more like that of the placenta tissue (Fig. 1A; Supplementary Fig. S1).

### The promoter of *aff* contains a large sequence deletion

**Fig. 2.**
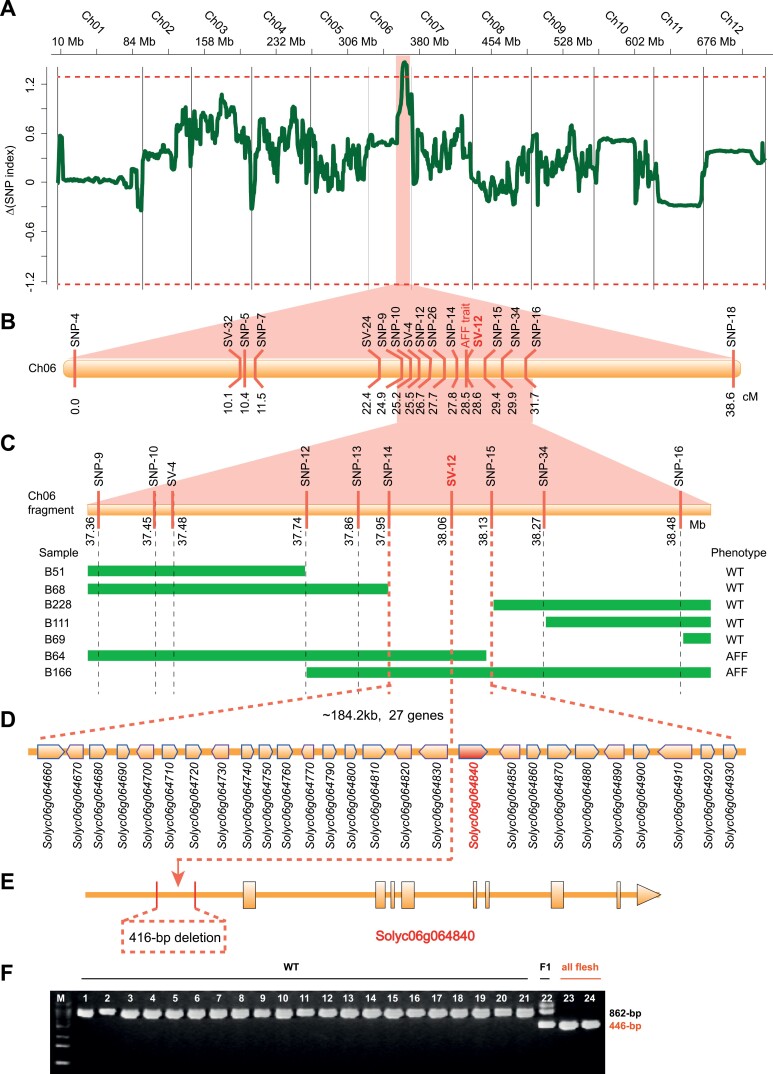
Map-based cloning of the *AFF* gene in tomato. (A) ∆(SNP index) as derived from bulked segregant analysis sequencing. The *x*-axis indicates the physical position on the tomato chromosomes whilst the *y*-axis shows the value of the SNP-index based on a 200-kb sliding window with a 20-kb increment. (B) Initial mapping of the *AFF* gene using 215 F_2_ plants derived from a cross between the *aff* line 06-790 and the wild-type (WT) LA4069. (C) Genotypes and phenotypes of homozygous recombinant plants derived from 249 BC_2_S_1_ plants generated by continued backcrossing of 06-790 to the WT H1706 (B51, B68, B228, B111, and B69 are normal lines; B64 and B166 are *aff* lines). (D) Annotated gene models in the mapping region according to the International Tomato Genome Sequencing Project version SL4.0 and annotation ITAG4.0 for H1706. (E) Gene structure of *AFF*. The 416-bp deletion in the *cis*-regulatory region of the gene is indicated. (F) PCR results for different varieties and lines using the marker SV-12 designed with the 416-bp deletion (shown in C). M, 100-bp DNA maker ladder.

Bulked segregant analysis sequencing (BSA-seq) was applied to locate the *AFF* gene. Using the genome sequence of *S. lycopersicum* (SL4.0 ITAG4.0) as the reference, we called out 298 942 SNPs that were polymorphic between P1 and P2 while being homozygous in the two parental genomes. These SNPs were then used in SNP index analysis (Takagi *et al.*, 2013) with the *aff* and the WT pools of the F_2_ population. We detected a significant signal (∆index=1.448, above the 99% confidence level) located between 37.25 Mb and 37.75 Mb on chromosome 6 (Fig. 2A), and 21 SNPs were located in this region. The mean SNP indexes in this region for the *aff* and WT pools were 0.98 and 0.32, respectively.

Linkage analysis of two populations (F_2_ with 215 individuals and BC_2_S_1_ with 249 individuals) was used to fine-map the *AFF* gene. Molecular markers were selected from these polymorphic SNPs and SVs between P1 and P2 (Supplementary Table S1), and were genotyped by PCR and KASP. The linkage signals of *AFF* overlapped with those of BSA-seq (Fig. 2B). With the genotype of these markers in the BC_2_S_1_ population, the *AFF* gene was finally mapped between markers SNP-14 and SNP-15 (SL4.0ch06: 37 945 500–38 129 705), which was ~184.2 kb and harbored 27 genes (Fig. 2C, D, Supplementary Table S3). A 416-bp deletion was found (SL4.0ch06: 38 062 128–38 062 543) located 1775 bp upstream of the gene *Solyc06g064840* (Fig. 2E), which contained no sequence polymorphisms in the coding sequences between *aff* and the WT. A marker named as SV-12 was designed with the 416-bp deletion and it showed complete co-segregation with all-flesh individuals (Fig. 2F). The gene associated with this deletion, *Solyc06g064840*, is a member of the AGAMOUS family belonging to the MADS-box D-class genes and is also named *SlMBP*3 (Zhang *et al*, 2019). The gene is specifically expressed in the developing locule (include the seeds) of tomato (Supplementary Figs S2, S3; Koenig *et al.*, 2013; Fernandez-Pozo *et al.*, 2017).

### Gene editing of *AFF* confirms its function in locule gel formation

**Fig. 3.**
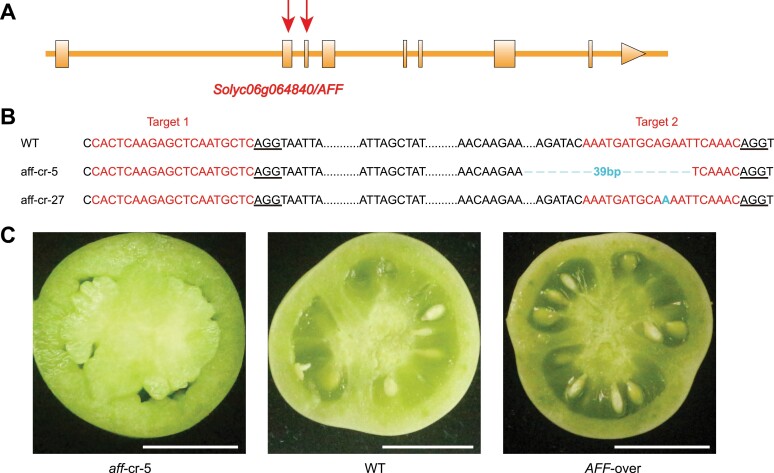
Characterization of CRISPR/Cas9-edited *aff* lines and *AFF*-overexpression lines. (A) Schematic diagram showing the two single-guide RNAs targeting the second and the third exon of *AFF*. (B) Sequences of the wild-type (WT) and two CRISPR/Cas9-edited *aff* mutants (*aff*-cr). The target sites of the single-guide RNAs are shown in red and protospacer-adjacent motif (PAM) sequences are underlined in black. Altered sequences in the edited lines are shown in blue. The *aff*-cr alleles were identified by cloning and sequencing PCR products of the *AFF-*targeted region from two T_0_ plants in the Micro Tom background. (C) Representative transverse sections of from the *aff*-cr-5 line, the WT, and the *AFF*-overexpression line at 25 d after flowering. The scale bars are 1 cm.

To confirm that *Solyc06g064840* is the causal gene of the *aff* genotype, we generated knock-out mutations using the CRISPR/Cas9 system with two sgRNAs that targeted the second and third exon of the *AFF* gene (Fig. 3A). These edited plants were confirmed by PCR amplification and DNA sequencing (Fig. 3B). We evaluated the first-generation (T_0_) diploid lines that were homozygous for the edited mutant alleles, and found that the *aff*-cr mutants produced the expected all-flesh fruits with normal seeds as in the WT but without locule gel (Fig. 3C). We also generated transgenic plants with overexpression of the *Solyc06g064840* (*AFF*) gene driven by the 35S promoter, and found that the T_1_ homozygous lines developed more locule gel compared with the Micro Tom WT fruit (Fig. 3C). These results indicated that *AFF* possessed the key function in locule gel formation in tomato fruit.

### The deleted promoter sequence is strongly conserved in Solanaceae

**Fig. 4.**
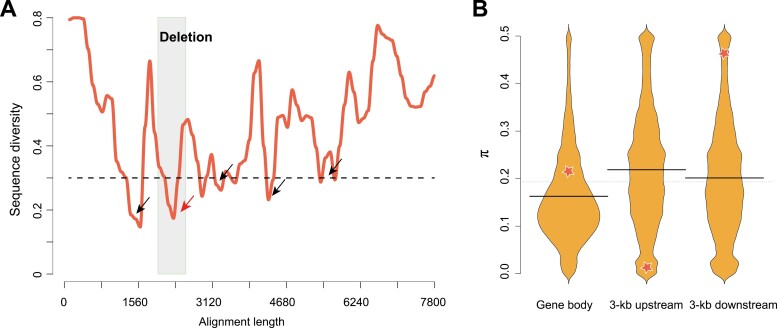
Sequence conservation of the *AFF* promoter in different Solanaceae species and within the tomato germplasm. (A) Sequence diversity of the promoter regions of orthologous *AFF* genes among five Solanaceae species (*Solanum lycopersicum*, *S. pennellii*, *S. tuberosum*, *S. melongena*, and *Capsicum annuum*). The location of the 416-bp deletion is indicated. The dashed horizontal line at diversity = 0.3 indicates the threshold used for identifying the conserved regions (indicated by the arrows). The conserved region located in the 416-bp deletion is highlighted by the red arrow. (B) Bean-plots of nucleotide diversity (π) values for the gene body and the adjacent regions 3-kb upstream and 3-kb downstream for all genes in the *S. lycopersicum* genome and for the *AFF* gene (stars). The 3-kb upstream region of *AFF* shows strong conservation compared with the other genes in the tomato germplasm.

To determine the detailed function of the 416-bp deletion, we analysed a 2-kb sequence including the deletion using the promoter prediction tool TSSP in the PlantProm (Shahmuradov *et al.*, 2003) and PlantCARE databases (Lescot *et al.*, 2002). We found that the 416-bp deletion in the promoter region of *Solyc06g064840* contained functional elements including the TATA box and CAAT box (Supplementary Table S4). We therefore decided to examine whether the 416-bp sequence was conserved across Solanaceae crop species. We selected two tomato genomes (*S. lycopersicum* and *S. pennellii*) together with potato (*S. tuberosum*), capsicum (*C. annuum*), and eggplant (*S. melongena*) and determined the syntenic orthologous genes of *AFF*, which were *Solyc06g064840*, *Sopen06g023350*, *Sotub06g020180*, *Capang01g002169*, and *Sme2.5_02049.1_g00007.1*, respectively. The promoter sequences of these five syntenic genes were extracted from their corresponding genomes and aligned using MUSCLE (Edgar, 2004), and we then estimated the conservation level of these promoter sequences based on the results of multiple sequence alignment. Using the nucleotide diversity π as the measure and a threshold of 0.3, we determined that five main local regions showed a relatively high conservation level in the promoter sequences of these Solanaceae crops (i.e. low mismatch ratio in multiple sequence alignment; Fig. 4A). These five regions should therefore have important roles in regulating the expression of associated genes. Moreover, the 416-bp sequence deletion was located at one of the two most-conserved regions. This suggested that the deletion might have a large effect in altering the expression of *AFF* in the *aff* genotype.

We further investigated the conservation of the promoter region within the tomato germplasm by analysing its sequence diversity using a previously published variome dataset of 360 tomato accessions (Lin *et al.*, 2014). We made a general estimate of the sequence diversity (selective sweep) by calculating π for the 3-kb upstream region, gene body, and 3-kb downstream regions for each of the 34 075 tomato genes and checked the selection strength, i.e. the diversity level of the *AFF* gene under the background of all tomato genes. The π value of the *AFF* gene body was 0.21, which was slightly higher than the mode value of all genes. Its downstream region had a π value of 0.46, which indicated higher diversity, whilst its upstream region had a π value of 0.026, which was less than 93.8% of all other genes (Fig. 4B). This suggested that the promoter region of *AFF* might undergo stronger selection pressure against mutations than many other genes in the tomato germplasm. Taken together, these findings indicated the importance of sequence conservation in the promoter region of *AFF*, which further suggested that the 416-bp deletion might have a significant impact on the function of the gene.

### The deletion in the promoter region down-regulates the expression level of *AFF*

**Fig. 5.**
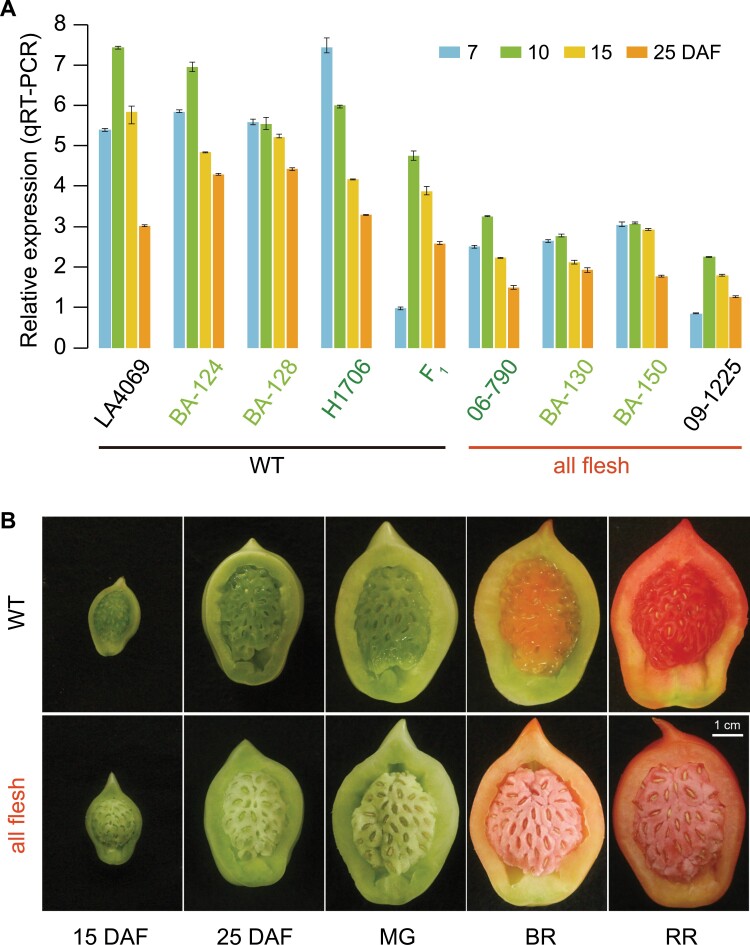
Expression of *AFF* in different wild-type and *aff* lines and the phenotypes of locule tissues at different development stages. (A) Relative expression of *AFF* transcripts in locule tissues at stages of development, as determined by qRT-PCR. *SlFRG27* (*Solyc06g007510*), *SlFRG03* (*Solyc02g063070*), and *ACTIN* (*Solyc11g005330*) were used as internal controls to normalize expression (Cheng *et al.*, 2017).WT, wild-type; DAF, days after flowering. BA-130 and BA-150 are *aff* lines derived from BC_2_S_1_ plants generated by the continued backcrossing of the *aff* line 06-790 to the WT H1706, and 09-1225 and 06-790 are all-flesh fruit cultivars. F_1_ indicates the F_1_ progeny of 06-790 crossed to H1706. H1706 and LA4069 are normal WT cultivars. BA-124 and BA-128 are normal lines. Data are means (±SD) of *n*=3 replicates. (B) Representative images of longitudinal sections of fruit locule tissue at different stages of development for the WT and an *aff* near-isogenic line created by backcrossing 06-790 to H1706 for six generations followed by two generations of selfing. MG, mature green; BR, breaker ripe; RR, red ripe.

**Fig. 6.**
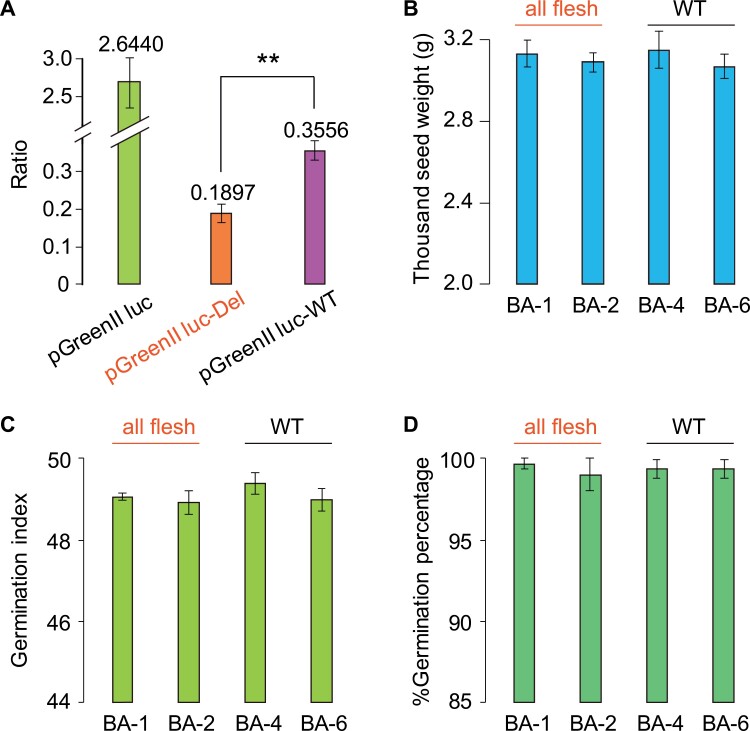
Transcriptional activity of the wild-type (WT) and *aff* promoters, and seed characteristics of WT and *aff* near-isogenic lines. (A) Results of dual-luciferase assays showing the LUC/REN ratio in leaves of *Nicotiana benthamiana* transformed with either the pGreenll vector with the *aff* promoter with the 416-bp deletion (pGreenll-LUC-Del) or the vector with the *AFF* wild-type promoter (pGreenll-LUC-WT); pGreenll-LUC was the blank vector with the 35S promoter. ; The significant difference was determined using Student’s *t*-test: **P<0.01. (B) Thousand-seed weight, (C) germination index (calculated as sum of germination per day), and (D) seed germination percentage of near-isogenic lines of the WT and *aff*. BA1-1 and BA2-1 are *aff* lines derived from BC_6_S_2_ plants generated by continued backcrossing of 06-790 to H1706, and BA4-1 and BA6-1 are normal lines derived from BC_6_S_2_ plants generated by continued backcrossing of 06-790 to H1706. All data are means (±SD) of *n*=3 replicates.

We investigated the expression of *AFF* by quantitative real-time PCR and the dual-luciferase reporter system. We first performed qRT-PCR analysis to determine the variation in expression of *AFF* at different developmental stages in locule tissues from four BC_2_S_1_ lines, together with their parental materials P1, H1706, and F_1_, as well as another *aff* line 09-1225 and the WT line P2 (LA4069). *AFF* was highly expressed in all of these samples at 7 DAF and 10 DAF, followed by a significant decrease at 15 DAF (Fig. 5A), which was synchronous with the differentiation of locule tissues and was consistent with previous studies (Koenig *et al.*, 2013; Fernandez-Pozo *et al.*, 2017). More importantly, the expression of *AFF* was significantly lower in *aff* samples than in WT samples. The *aff* BC_2_S_1_ lines BA-130 and BA-150 had a significantly lower expression of *AFF* than the WT BC_2_S_1_ lines BA-124 and BA-128. We then evaluated the transcriptional activity of the promoter sequences in the WT and *aff* samples using the LUC/REN dual-luciferase reporter system, and found that the relative activity of LUC in the *aff* promoter with the 416-bp deletion was significantly lower than that of the WT promoter (Fig. 6A).

We examined the locule tissues of *aff* and WT fruits using near-isogenic lines (NILs). These were generated by back-crossing the *aff* tomato lines 06-790 to H1706 for six generations, assisted by molecular selection of the marker SV-12. These *aff* lines all produced all-flesh fruit in which the locule tissues maintained a solid state during development, which was distinct from the WT line H1706 (Fig. 5B). We also examined seed characteristics, and found that the appearance of the seeds did not differ between the *aff* and the WT lines (Supplementary Fig. S4). The *aff* lines BA-1 and BA-2 had similar thousand-seed weights, germination indexes (calculated as the sum of germination per day), and germination rates to those of the WT lines BA-4 and BA-6 (Fig. 6B–D). These results suggested that the deletion in the promoter stops the formation of gel in tomato fruit but does not affect the function of *SlMBP3*/*AFF* in relation to the normal development of seeds. The effect of the deletion mutation in the *aff* plants was different from that of the *aff*-cr plants that could not produce seeds (Fig. 3C), and from *SlMBP3*-RNAi plants whose seeds are not able to germinate (Zhang *et al.*, 2019).

### The *aff* mutation has widespread effects on gene expression and metabolic components

We compared whole-genome gene expression patterns between HZ106 (WT) and its NIL BA-1 (*aff* line) with the *AFF* gene replaced by the mutated one with the 416-bp deletion in its promoter region. mRNA-seq analyses were performed on the locule and placenta for the WT and *aff* line at three time-points (10, 15, and 25 DAF). In the differentially expressed gene (DEG) sets between the WT and *aff*, we found enrichment of genes belonging to Gene Ontology (GO) terms including lipid metabolism, plant-type cell wall, phytohormones, metabolism and catabolism, flavonoid biosynthesis, glucosyltransferase activity (Supplementary Table S5), and in KEGG pathways including sugar metabolism and phytohormone biosynthesis (Supplementary Table S6). Among the top 50 enriched GO terms, 1110 genes were down-regulated and only 359 were up-regulated (Supplementary Table S5). There were 55 DEGs in the KEGG pathway ‘MAPK signaling’, of which 42 were down-regulated and 13 up-regulated. The reduced expression of *AFF* in the *aff* line was thus clearly associated altered expression of a large number of genes, most of which were down-regulated.

**Fig. 7.**
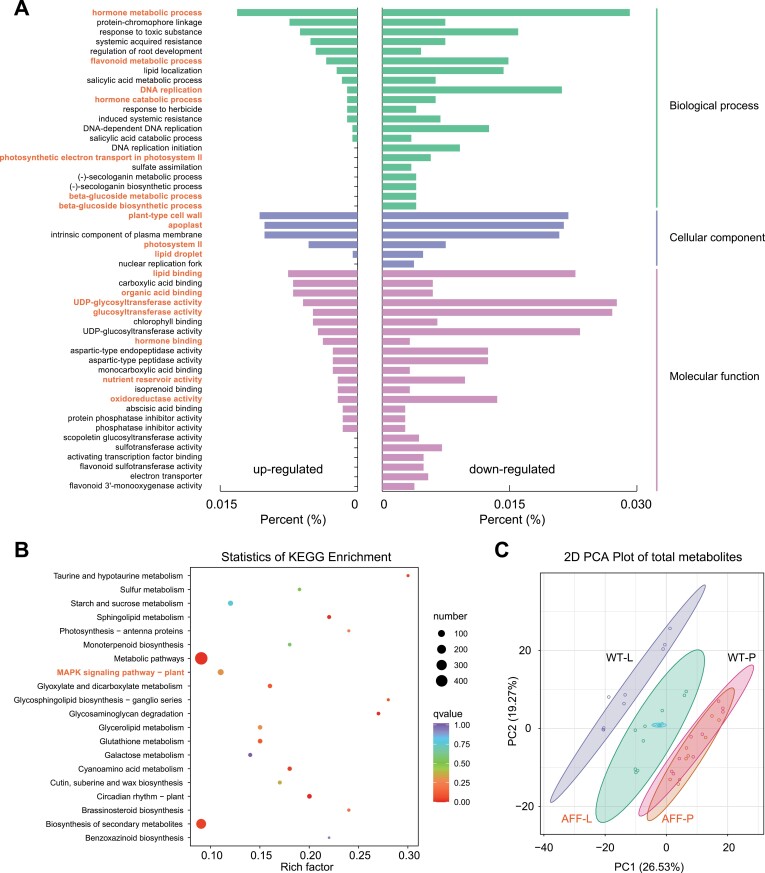
Differential patterns of gene expression and metabolite relative contents between all-flesh fruit and wild-type tomatoes. (A) Significantly enriched GO terms and (B) KEGG pathways of differentially expressed genes in the locule tissues between the near-isogenic *aff* line BA-1 and the wild-type (WT) HZ106. The names of terms and pathways that are considered to be associated with locule development are highlighted. (C) Principal component analysis (PCA) of metabolite relative contents in the locule (L) and placenta (P) tissues of the WT and the *aff* line.

We performed detailed pair-wise comparisons between the transcriptome datasets. When comparing expression between the locule and placenta tissues in the WT, we found enrichment of DEGs included in the GO terms lipid transport, apoplast, flavonoid metabolism, transferase, and hydrolase activity, and the KEGG pathways metabolism, protein kinase, and phytohormone (Supplementary Fig. S5). However, the GO terms lipid transport and flavonoid metabolism were not enriched in DEGs between the locule and placenta tissues in *aff*, and this comparison also featured over-representations of GO terms for phloem and xylem, as well as symporter activity and transmembrane transmission-related (Supplementary Fig. S6). Focusing on DEGs in the locule between the WT and *aff*, we found enrichment in GO terms and KEGG pathways that were similar to those observed in the comparison between the locule and placenta tissues in the WT (Fig. 7A, B). In addition, the GO terms DNA replication, plasma membrane, photosystem II, plant-type cell wall, glucosyltransferase activity, and nutrient reservoir activity were specifically enriched in this group (Supplementary Table S7, Fig. 7A). In contrast, none of these GO terms and KEGG pathways were enriched in the DEGs in the placenta tissue between the WT and *aff* (Supplementary Fig. S7). Overall, these results suggested that reduced expression of *AFF* was associated with down-regulation of expression of genes involved in DNA replication, phytohormone metabolism, photosynthesis, sugar metabolism, and MAPK signaling, which in turn might then have prevented the liquefaction process in the locule that normally occurs in WT tomato.

Genes involved in hydrolases, phytohormone metabolism, and DNA replication have been reported to control liquefaction of the locule tissue in tomato fruit (Huber and Lee, 1986; Mounet *et al.*, 2009; Chevalier *et al.*, 2014; Takizawa *et al.*, 2014; Uluisik *et al.*, 2016). We found that a total of 188 genes showed strong and stable differential expression between the WT and *aff*, of which 122 were down-regulated and 66 were up-regulated in *aff* (Supplementary Table S8). Many of these DEGs were related to the locule tissue-liquefaction process. First, six genes related to gibberellins were down-regulated in *aff*, of which four were gibberellin-regulated proteins, one was involved in the gibberellin biosynthesis process, and the other was involved in the gibberellic acid-mediated signaling pathway (Supplementary Table S8). We also found six DEGs related to auxin, of which the auxin repressed protein was up-regulated in *aff*, while the other five were down-regulated, namely auxin transporters, auxin responsive protein IAA9, auxin-related genes from the GH3 family, and those involved in the auxin signaling pathway Second, there were three copies of cytochrome P450 genes with expression down-regulated in *aff* (Supplementary Table S8), indicating a low level of energy-related activity in *aff*. Third, pectinesterase, which is strictly regulated and functions in the softening of tomato fruit, was greatly down-regulated in *aff*, which could clearly have an important impact on the solidity of the fruits. Fourth, we identified two copies of genes related to xyloglucan endo-transglycosylase (XET) that were down-regulated in *aff*. XET is involved in the induction of fruit ripening and softening, and its down-regulation would be expected to hinder the softening of fruits. Fifth, we found three copies of glycosyl hydrolase genes that were down-regulated in *aff*, indicating suppressed metabolism of glycolysis compared with the WT. More importantly, we found that *TAG1* (*Solyc02g071730*) and *TAGL1* (*Solyc07g055920*), both of which are paralogs of *AFF* and show high levels of sequence homology (Supplementary Fig. S8), were up-regulated in the locule tissue of *aff* compared with the WT. However, *TAGL11* (*Solyc11g028020*), which is the paralog with the highest homology to *AFF* in tomato (Huang *et al.*, 2017), showed no difference in expression between *aff* and the WT.

Our examination of the metabolomes in the fruits of the WT and *aff* line supported the results of the mRNA-seq analysis. Principal component analysis showed that the placenta tissues from both genotypes had similar metabolic components (Fig. 7C). In contrast, the metabolites in the locule tissue differed between the WT and *aff*, and both were different to the placenta. The pattern of locule metabolites in *aff* was intermediate between that of the placenta and locule tissues of the WT, indicating that the down-regulation of expression of *AFF* changed the metabolic components of the tissue. We further examined the differential metabolites and found higher relative contents of flavonoids and lipids in *aff*, whereas there were more alkaloids and phenolic acids in the WT (Supplementary Table S9). Overall, the results indicated that the distinct fruit qualities of the *aff* tomato fruit were caused by down-regulated expression of *AFF* and associated large-scale variations in gene expression.

## Discussion

Locule gel liquefaction is a significant process in development and ripening and is also a typical characteristic of tomato fruit. In this study, we identified the causal gene of the all-flesh fruit trait, *AFF*, and the 416-bp deletion mutation in the *cis*-regulatory region of the gene (Fig. 2). We found that the expression dosage of *AFF* was crucial for locule tissue liquefaction, and this was consistent with the functional characterization of *AFF*/*SlMBP3* reported in a recent study (Zhang *et al.*, 2019). *AFF* belongs to the AGAMOUS subfamily and contains a typical MADs-box domain; its paralogous genes in tomato are *TAG1*, *TAGL1*, and *TAGL11*, which have a high sequence homology to each other (Supplementary Fig. S8). These genes and their orthologs have been found to play important roles in ovule differentiation and formation, to participate in seed and coat development, and to control the expansion and ripening processes of the carpel and fleshy fruit in many species (Angenent *et al.*, 1995; Colombo *et al.*, 1995; Favaro *et al.*, 2003; Vrebalov *et al.*, 2009; Itkin *et al.*, 2010; Pan *et al.*, 2010; Ocarez and Mejía, 2016; Zhang *et al.*, 2019).

*AFF* plays a major role in locule liquefaction, and its function probably cannot be compensated for by its paralogs *TAG1*, *TAGL1*, or *TAGL11.* The *cis*-regulatory sequence deletion mutation of the *AFF* gene was associated with differential expression of many important genes (SupplementaryTable S8). Among them, we observed that the expression of *TAG1* and *TAGL1* was significantly up-regulated, whilst the other paralog, *TAGL11*, which functions in early fleshy fruit development in tomato (Huang *et al.*, 2017), showed stable expression between *aff* and the WT. Given that this did not recover the development of normal liquefied locule tissue in the *aff* lines, the function of mediating locule tissue liquefaction is likely to mainly belong to *AFF*, although *TAGL11* has been shown to have some functional redundancy with *AFF* in seed development of tomato (Huang *et al.*, 2017; Zhang *et al.*, 2019).

Based on our metabolomics analysis, we found that the pattern of locule metabolites in *aff* was intermediate between that of the placenta and locule tissues of WT fruit (Fig. 7C). This indicated that the tomato locule tissue is derived from the placenta, which is formed from the development of the carpel (Davies and Cocking, 1965; Gallego *et al.*, 1991; Lemaire-Chamley *et al.*, 2005). The carpel development process is regulated by D-class genes in the ‘ABCDE’ flower development model, which is consistent with the fact that the locule gel develops along with the degradation of the cell wall matrix (Brecht, 1987; Joubès *et al.*, 1999; Lemaire-Chamley *et al.*, 2005).

The reduced expression dosage of *AFF* caused by the 416-bp *cis*-regulatory deletion was the key factor that promoted the formation of the AFF trait (Fig. 5). The dosage of gene expression has been proved to play an important role in the variation of plant traits, especially for floral organ identity. Up- or down-regulation of the expression of a single ABCDE-class gene can easily shift the boundaries between different types of floral organs (Ito *et al.*, 2007; Wuest *et al.*, 2012; Wang *et al.*, 2016). For example, a dosage imbalance between B- and C-class proteins can change stamen morphology in *Petrocosmea* (Liu *et al.*, 2018), while variation in expression of *TAGL1* and *TAGL11* can also affect the development of tomato seeds and the fleshy characteristics of fruits (Vrebalov *et al.*, 2009; Gimenez *et al.*, 2016; Ocarez and Mejía, 2016; Huang *et al.*, 2017). In addition to genes specifically related to floral determination, Rodriguez-Leal *et al*. (2017) showed that gene-editing of different loci in the promoter region of other tomato genes results in the formation of fruits with different sizes. Structural variants (SVs) have been found to be a major genetic resource that can be used to create dosage variations in gene expression (Alonge *et al.*, 2020). Unlike SNPs, the SVs located in *cis*-regulatory regions of genes always cause changes in the expression dosage and hence produce genetic and phenotypic changes. SVs have been reported to be involved in the formation of many traits in plants, and play an important role in plant evolution and crop domestication (Rodriguez-Leal *et al*., 2017; Lye and Purugganan, 2019; Alonge *et al.*, 2020). In our study, a 416-bp sequence deletion—a type of SV—within the *cis*-regulatory region of *AFF* down-regulated its expression and the resulting dosage effect produced the all-flesh fruit trait (Fig. 5).Thus, SVs are potentially useful quantitative variants that could be used in next-generation breeding strategies through genetic engineering in the future (Swinnen *et al.*, 2016; Rodriguez-Leal *et al*., 2017; Alonge *et al.*, 2020).

The variation in *AFF* might also have contributed to the evolution of fleshy fruit in Solanaceae, and hence it might provide insights into the evolution of fruit types in plants. Evidence obtained through paleobotany and molecular biology has shown that fleshy fruit plants evolved from dry fruit plants, but the molecular mechanisms responsible for the shift from remain unknown (Seymour *et al.*, 2008; Kumar and Khurana, 2014; Maheepala *et al.*, 2019). Determining these mechanisms and their underlying genetic basis is critical for understanding the evolution of biodiversity; however, the lack of intermediate or transition fruit-types has limited research progress in this area (Becker *et al.*, 2011; Wang *et al.*, 2015). Comparative genetic analysis has shown that there are widespread genomic synteny and collinearity of genes among Solanaceae species, especially in vegetable crops (potato, tomato, capsicum, and eggplant), the fruits of which show similar characteristics but are also varied in many aspects. These differences in fruit development could be caused by differences in gene expression or by sequence variations of similar genes (Kim *et al.*, 2014). For example, there is more locule gel in the wild tomato *S. lycopersicum* var. *cerasiforme* and in *S. pimpinellifolium* than in cultivated tomato (Lemaire-Chamley *et al.*, 2005) and this might suggest a positive relationship between the quantity of liquefied locule tissues and the expression level of the *AFF* gene through the process of tomato domestication and breeding.

In summary, the phenotype of *aff* tomato, in which the locule tissue is changed from a jelly-like substance to a solid state, was found to be caused by a structural variant consisting of a 416-bp sequence deletion in the *cis*-regulatory region of the *AFF* gene. This SV mutation reduced the expression dosage of *AFF*, which then affected the normal liquefication process of the locule tissue through altered expression of many important genes and consequent changes in the metabolic components of the fruit. Our findings provide important information on the mechanisms that underly the changes that occur inside developing tomato fruit and shed new light on the evolution of berry fruits. Future systematic studies on the dosage effects of *AFF* expression and extensive examination of the formation and development processes of fruit locule tissues should help to reveal the evolutionary mechanism of berry fruits.

## Supplementary data

The following supplementary data are available at *JXB* online.

Table S1. Markers used for fine-mapping and sequencing of the 416-bp deletion.

Table S2. Primers used in this study.

Table S3. The 27 genes identified in fine-mapping region.

Table S4. The *cis*-element motifs of the 416-bp deletion sequence.

Table S5. Significantly enriched GO terms of differentially expressed genes between the wild-type and all-flesh fruit tomato.

Table S6. Significantly enriched KEGG pathways of differentially expressed genes between the wild-type and all-flesh fruit tomato.

Table S7. Significantly enriched GO terms of differentially expressed genes in locule tissues between the wild-type and all-flesh fruit tomato.

Table S8. Differentially expressed genes in the placenta and locule tissues of the wild-type and all-flesh fruit tomato at different stages of development.

Table S9. Metabolites showing significantly different relative contents in the placenta and locule tissues of the wild-type and all-flesh fruit tomato at different stages of development.

Fig. S1. Longitudinal sections of fruit of the wild-type and all-flesh fruit tomato.

Fig. S2. The expression of *AFF* in different organs of whole plants of M82 and LA0716 based on the data of Koenig *et al.* (2013).

Fig. S3. Heatmap of the expression of *AFF* in different fruit tissues at different stages of development in M82 tomato.

Fig. S4. Representative images of seed germination of all-flesh fruit tomato NILs.

Fig. S5. Functional annotation of differentially expressed genes between locule and placenta tissues of wild-type tomato.

Fig. S6. Functional annotation of differentially expressed genes between locule and placenta tissues of all-flesh fruit tomato.

Fig. S7. Functional annotation of differentially expressed genes in placenta tissues between the wild-type and all-flesh fruit tomato.

Fig. S8. Phylogenetic tree of AFF and homologous AGAMOUS proteins in tomato and other closely related species.

erab401_suppl_Supplementary_S1-S4_Figures_S1-S8

erab401_suppl_Supplementary_Tables_S5-S9

## Data Availability

The mRNA-seq and metabolic data are openly available at http://www.bioinformaticslab.cn/files/tomato_AFF/.

## Abbreviations

AFF: all-flesh fruit
NIL: near-isogenic line
WT: wild-type
SV: structural variant
KASP: Kompetitive allele specific PCR
π: nucleotide diversity
DAF: days after flowering
PCA: principal component analysis
HCA: hierarchical cluster analysis
