# Supplementary material for: All-flesh fruit in tomato is controlled by reduced expression dosage of AFF through a structural variant mutation in the promoter
Source: J Exp Bot. 2021 Sep 7;73(1):123–38. doi: 10.1093/jxb/erab401 (PMC8730696; doi:10.1093/jxb/erab401)
Supplement: erab401_suppl_Supplementary_S1-S4_Figures_S1-S8 [file erab401_suppl_supplementary_s1-s4_figures_s1-s8.pdf]

All-Flesh Tomato Regulated by Reduced Expression Dosage of *AFF* Through a Promoter SV Mutation

Lei Liu[1], Kang Zhang[1], JinRui Bai, Jinghua Lu, Xiaoxiao Lu, Junling Hu, Chunyang Pan, Shumin He, JialeYuan, Yiyue Zhang, Min Zhang, Yanmei Guo, Xiaoxuan Wang, Zejun Huang, Yongchen Du, Feng Cheng*, Junming Li*

Key Laboratory of Biology and Genetic Improvement of Horticultural Crops of Ministry of Agriculture, Institute of Vegetables and Flowers, Chinese Academy of Agricultural Sciences, Beijing 100081, China

[1] These authors contributed equally to this article.

*Correspondence: Junming Li (lijunming@caas.cn) and Feng Cheng (chengfeng@caas.cn)

This PDF file includes:

Supplementary Tables S1 to S4

Supplementary Figs. S1 to S8

Legends for Supplementary Tables S5-S9

Supplementary Tables

Table S1. Markers Used for Fine Mapping and Sequencing of the 416-bp Deletion.

Marker ID	Position (Sl4.0)	Sequence	Type
SNP-4	ch06: 35412148	TAATTTAACCTTATT[T/C]GTATCTTTAATATT	KASP
SNP-5	ch06: 36363062	TCTACTCCACTGCAC[C/T]CAAATGGATAAGTT	KASP
SNP-7	ch06: 36885620	TCTTGTTATGACATA[G/A]TTTGAATATATTAA	KASP
SNP-9	ch06: 37364332	CTTCATTGACCGCTA[G/A]ATCACACTTATAGA	KASP
SNP-10	ch06: 37453835	TTTGATTTTGTATAG[C/T]CTTTCTCGTGAACT	KASP
SNP-12	ch06: 37744940	AGGTTTGGCGAAAGA[C/A]GAATGACTTCACGC	KASP
SNP-13	ch06: 37857325	TGAGATGAGTAGAGT[C/T]ACCTTTTAGGAAAT	KASP
SNP-14	ch06: 37945500	CAGGATTTCAGGCAG[C/T]ATATATCTTGTATT	KASP
SNP-15	ch06: 38129705	CATTCTTACTCAAAA[T/A]TTTTTTAATGATGT	KASP

SNP-16	ch06: 38484067	ATAATTTATTTATTT[T/A]TTTTGTGTAGGGTC	KASP
SNP-18	ch06: 41189907	CTGGAATAATTCCGT[C/T]CCTCCCTTGATTTT	KASP
SNP-26	ch06: 37860753	TTCAGAATTTTTTTT[A/T]AGCTATTTATATGC	KASP
SNP-34	ch06: 38272907	CTTCTTTAAAAAATA[G/A]AAGGGAAAAAAGGC	KASP
SV-4	ch06:37484163-37484300	CCTACAAGAATACAAAGCCT<br>GGATGGCTCTAATGACAATG	PCR
SV-12	ch06:38062128-38062543	CCGTTTGAATAGGTTTAGTAGTCG<br>CTGAAAACTCACACAACTTCCAAA	PCR
SV-24	ch06:37134850 - 37135324	AGAGATTGCGTAGTAGTCAGCG<br>CTGGACCTGTTGGGACACTTA	PCR
SV-32	ch06:36573534-36573665	CCCCTTAAACCTCCCTCATA<br>ATCGCCACTAATGCCTTGA	PCR
AFF	*Solyc06g064840*	TATTTTTAATTTAAAAGCTATTTTTTTAAGCCAATCCAGACGGTCTCTTAATATACAGGTCAAACCTCATTAAATAAAATTTAAATATTTGAAAGAAAAGTTTGAGAGATTTTAAACAGCACAAGGGGCATATTAGTCAAGAAGAAACAAAAATAACACGCTTTGCAATAATTGGTGAAATTTTAGTCTGCAATAAACAATCCCATAACATCACGTCTGGTTTATATCTGGAAAAAAGCCATTTGAATGTCATTTTCTTGG	416-bp deletion

	CCAGCCATCTCTATTATCTCTCTTCACTTTAATTTTGAGTGATACTTTCTTCGTCCATCCGACTCAACACACATCTTTTAAGAAATAATAAATTCGAAGAGTAATTTTATTATATATCATCAGTCACCCCTATTGGTAACACGTCATCTAAATAT

Table S2. Primers Used in this Study.

Gene name	Gene ID	Sequence	Application
AFF	*Solyc06g064840*	GCATCTGGTTGGTGAAGG<br>ATCTGATTCTGCTGATGCC	Quantitive RT-PCR
SlFRG03	*Solyc02g063070*	GGCTGAACTGGCTCCTACTC<br>TTTCGCAAGGTTACAAGCAC	Quantitive RT-PCR
SlFRG27	*Solyc06g007510*	CTCTCTGTTGACAGACCCA<br>GAGTCCAGCTACGAGCAGTG	Quantitive RT-PCR
ACTIN	*Solyc11g005330*	GGAGATTGAAACTGCCAGGAGCA<br>CTGCAGCTTCCATACCAATCATGG	Quantitive RT-PCR
AFF	*Solyc06g064840*	TGATTGTGAGTGGTGCAAGCATTAGGTTT<br>TCTAAAACCTAATGCTTGCACCACTCACA	CRISPR-Cas9 Genome-editing
AFF	*Solyc06g064840*	TGTAGTTTGCTGCATCATTTGTATCTGT<br>AAACACAGATACAAATGATGCAGCAAAC	CRISPR-Cas9 Genome-editing
AFF	*Solyc06g064840*	TCAGCAGTCGAAGAGCTTTTTGCAGATTCTGAAATG<br>TTAGCGTGTGAAGAGCCTGAAGTTCAAGGGACTTCTTC	Over-expression

Table S3. The 27 Genes Identified in Fine Mapping Region.

Gene ID	Description（SGN）4.0
Solyc06g064660	Cyclin-dependent kinase C-1
Solyc06g064670	NADP-dependent alkenal double bond reductase P1
Solyc06g064680	NBS-coding resistance gene analog
Solyc06g064690	NBS-coding resistance gene analog
Solyc06g064700	Nucleoporin autopeptidase
Solyc06g064710	NB-LRR tospovirus immune receptor
Solyc06g064720	NB-LRR tospovirus immune receptor
Solyc06g064730	Nucleoporin autopeptidase
Solyc06g064740	nuclear nucleic acid-binding protein C1D
Solyc06g064750	Reistance protein F
Solyc06g064760	Reistance protein F
Solyc06g064770	Unknown protein
Solyc06g064790	Disease resistance protein
Solyc06g064800	Glucuronoxylan 4-O-methyltransferase 2
Solyc06g064810	zinc finger/BTB domain protein
Solyc06g064820	GDSL esterase/lipase
Solyc06g064830	WD-40 repeat protein AY032884
Solyc06g064840	AGAMOUS-like MADS-box transcription factor
Solyc06g064850	Protein yippee-like
Solyc06g064860	P-loop containing nucleoside triphosphate hydrolases superfamily protein
Solyc06g064870	Methyl esterase 12
Solyc06g064880	Nitrilase family protein (Carbon-nitrogen hydrolase)
Solyc06g064890	Transcription initiation factor IIE subunit beta
Solyc06g064900	Unknown protein
Solyc06g064910	T-complex 11
Solyc06g064920	P-loop containing nucleoside triphosphate hydrolases superfamily protein
Solyc06g064930	P-loop containing nucleoside triphosphate hydrolases superfamily protein

Table S4. The Cis-element Motifs of the 416-bp Deletion Sequence.

Putative cis-element motifs	Sequence	Location	Function
AAGAA-motif	GAAAGAA	+90	
AE-Box	AGAAACAA	+142	part of a module for light response
ARE	TGGTTT	+217	cis-acting regulatory element essential for the anaerobic induction
ATC-motif	GCCAATCC	+29	part of a conserved DNA module involved in light responsiveness
Box I	TTTCAAA	-87	light responsive element
Box-W1	TTGACC	-57	fungal elicitor responsive element
CAAT-box	CCAAT/CAAT/ CAATT/CAAAT	+30，-190，-169，-240，-86，+197，-170，-392，+31，+165	common cis-acting element in promoter and enhancer regions
CGTCA-motif	CGTCA	+402	cis-acting regulatory element involved in the MeJA-responsiveness
G-box	CACGTC	+211，+400	cis-acting regulatory element involved in light responsiveness
GAG-motif	AGAGATG	-266	part of a light responsive element
HD-Zip	CAAT(A/T)ATTG	+165	element involved in differentiation of the palisade mesophyll cell
O2-site	GATGA(C/T)(A/G) TG(A/G)	-202，-399	cis-acting regulatory element involved in zein metabolism regulation
Skn-1_motif	GTCAT	+248，+403	cis-acting regulatory element required for endosperm expression
TATA-box	TTTTA/TAATA/ TATA/TATTTAAA/ TATAAA/TATAA/ ATTATA/TATATAA/ ATATAT	+3，-11，+23，+48，+51，-74，-79，+109，-130，+180，	core promoter element around -30 of transcription start

		-220，-221，-222，-272，+336，<br>+346，+365，-368，+369，-370，<br>-371，-372，-373	
TGACG-motif	TGACG	-402	cis-acting regulatory element involved in the MeJA-responsiveness
W box	TTGACC	-57	

Table S5. Significantly Enriched GO Terms of Differentially Expressed Genes Between the Wild-Type and All-Flesh Fruit Tomato.

(Excel spreadsheet**)**

Table S6. Significantly Enriched KEGG Pathways of Differentially Expressed Genes Between the Wild-Type and All-Flesh Fruit Tomato.

(Excel spreadsheet**)**

Table S7. Significantly Enriched GO Terms of Differentially Expressed Genes in Locule Tissues Between the Wild-Type and All-Flesh Fruit Tomato.

(Excel spreadsheet**)**

Table S8. Differentially Expressed Genes in the Placenta and Locule Tissues of the Wild-Type and All-Flesh Fruit Tomato at Different Stages of Development.

(Excel spreadsheet**)**

Table S9. Metabolites Showing Significantly Different Contents in the Placenta and Locule Tissues of the Wild-Type and All-Flesh Fruit Tomato at Different Stages of Development.

(Excel spreadsheet**)**

Supplementary Figures

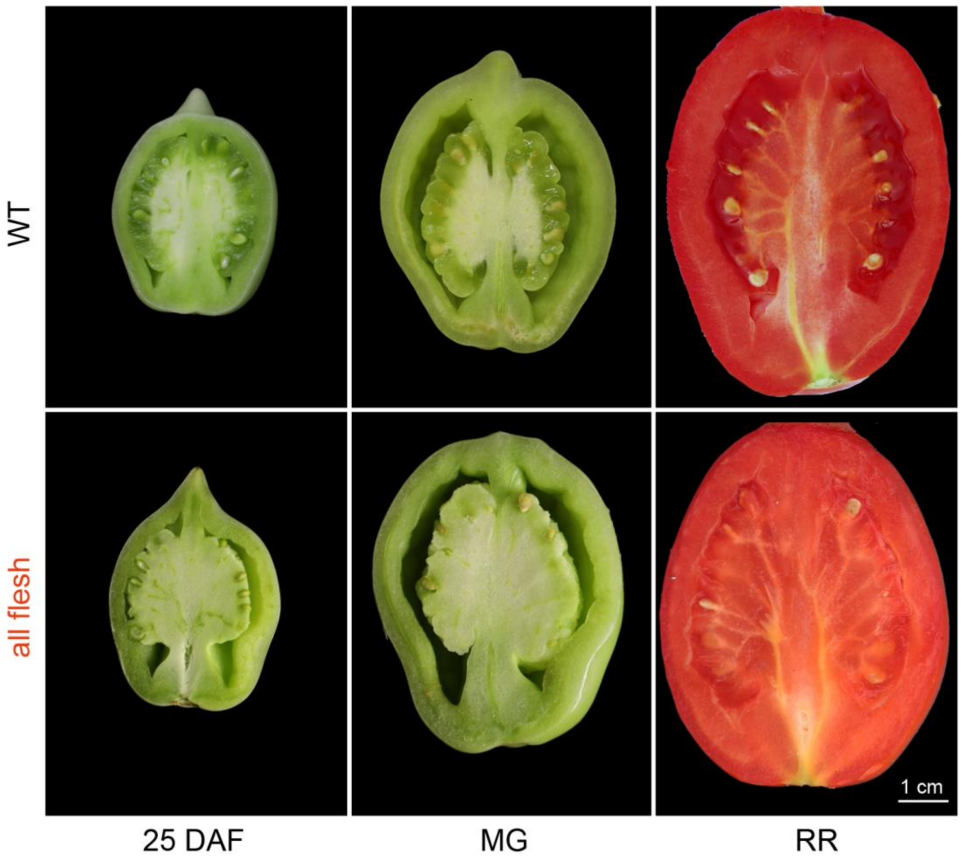

Fig. S1. Longitudinal Sections of Fruit of the Wild-Type and All-Flash Fruit Tomato. The appearance of locule tisues at three developmental stages of WT tomato LA4069 and *aff* tomato 06-790. DAF: days after flowering; MG: mature green; RR: red ripe.

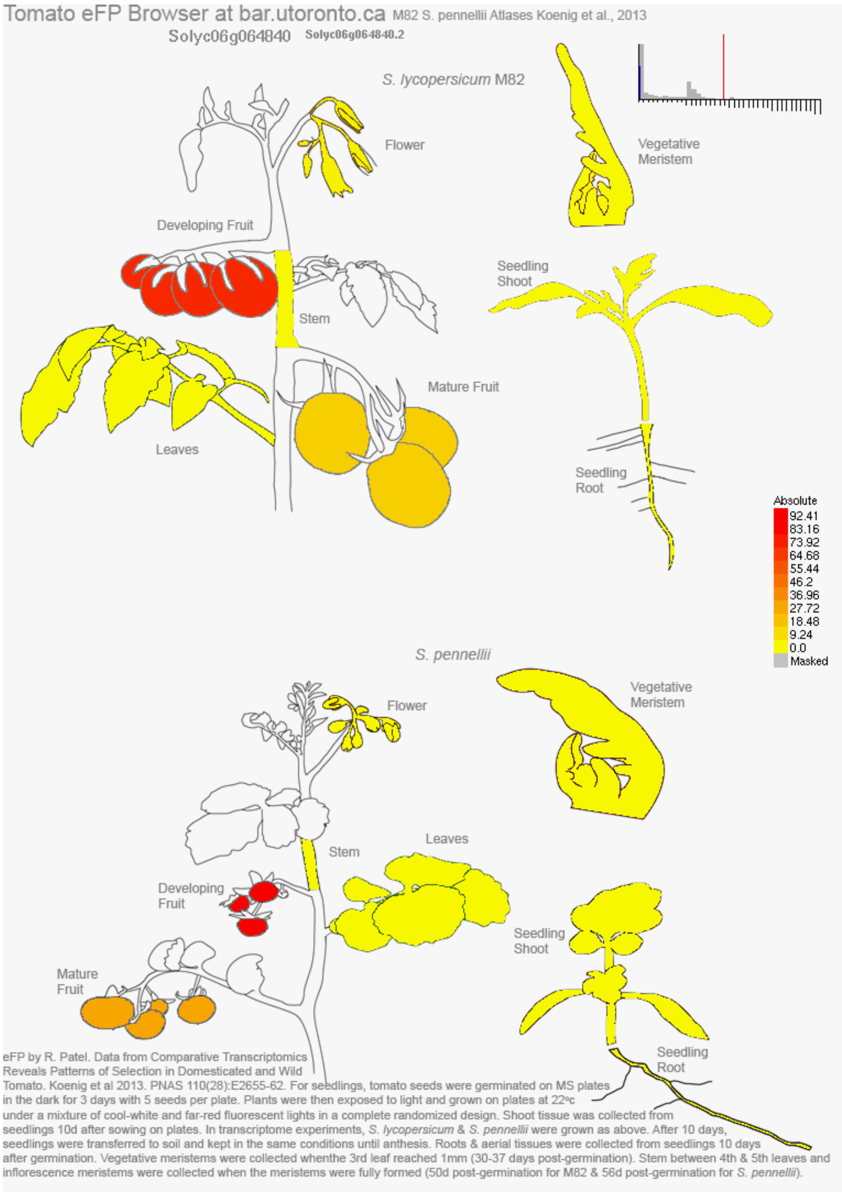

Fig. S2. The Expression of *AFF* in Different Organs of Whole Plants of M82 and LA0716 based on the data of Koenig *et al*. (2013)**.** Above is the expression level of *AFF* gene in different organs of M82 (*S. lycopersicum*), whereas the below one is the expression level of *AFF* gene in different organs of LA0716 (*S. pennellii*).

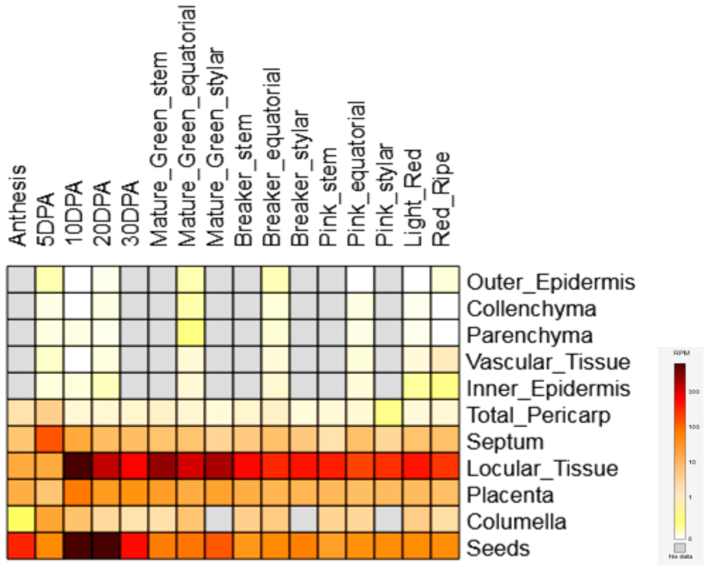

Fig. S3. Heatmap of the Expression of *AFF* in Different Fruit Tissues at Different Stages of Development in M82 Tomato, Displayed by SGN TEA (Fernandez-Pozo *et al*., 2017, Bioinformatics)**.** Longitudina axis represents different tissue of fruit. Transverse axis represents different developmental stages of fruit. DPA: day after anthesis.

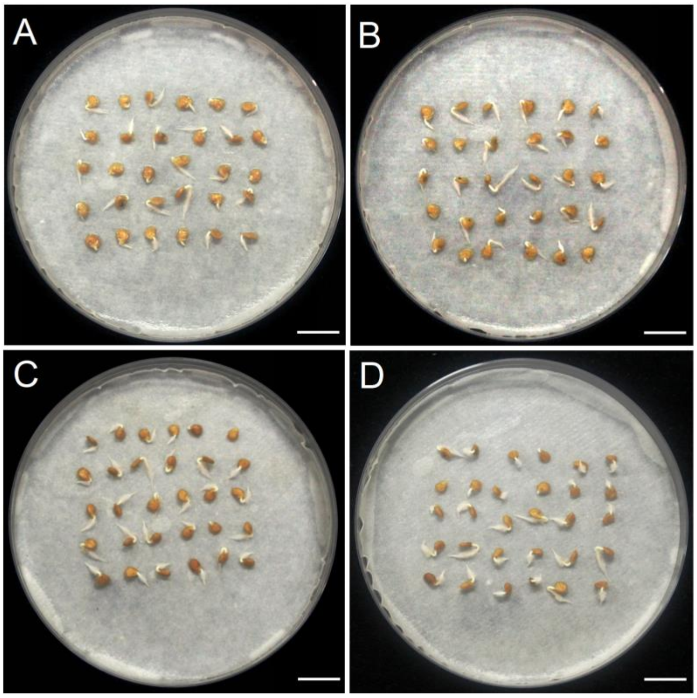

Fig. S4. Representative images of Seed Germination of All-Flesh Fruit Tomato NILs. The third day germination of seeds of *aff* NILs, *aff* genotype: BA-1 (**A**), BA-2 (**B**); WT type: BA-4 (**C**), BA-6 (**D**). Scale bars: 1 cm.

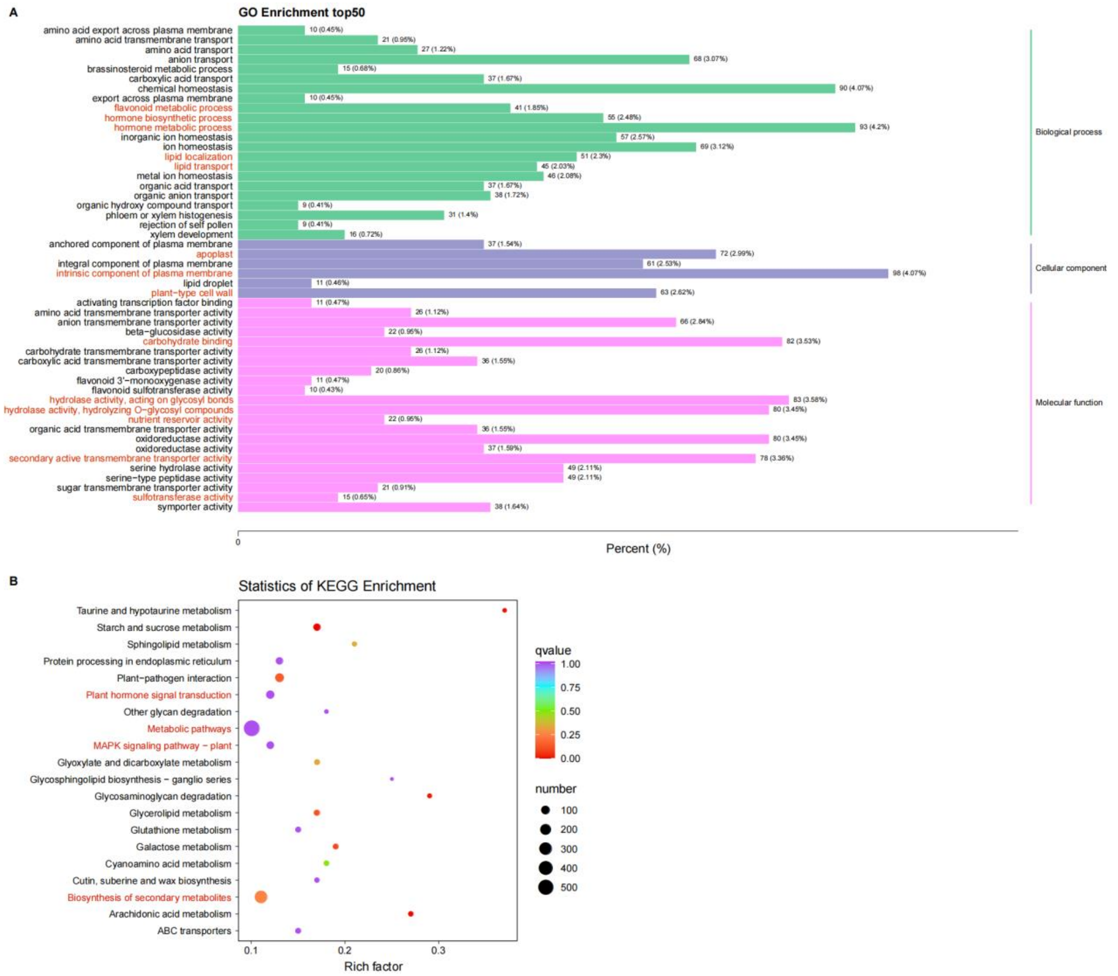

Fig. S5. Functional Annotation of Differentially Expressed Genes Between Locule and Placenta Tissues of the Wide-Type Tomato. (**A**) The significantly enriched GO terms; (**B**) The significantly enriched KEGG pathways.

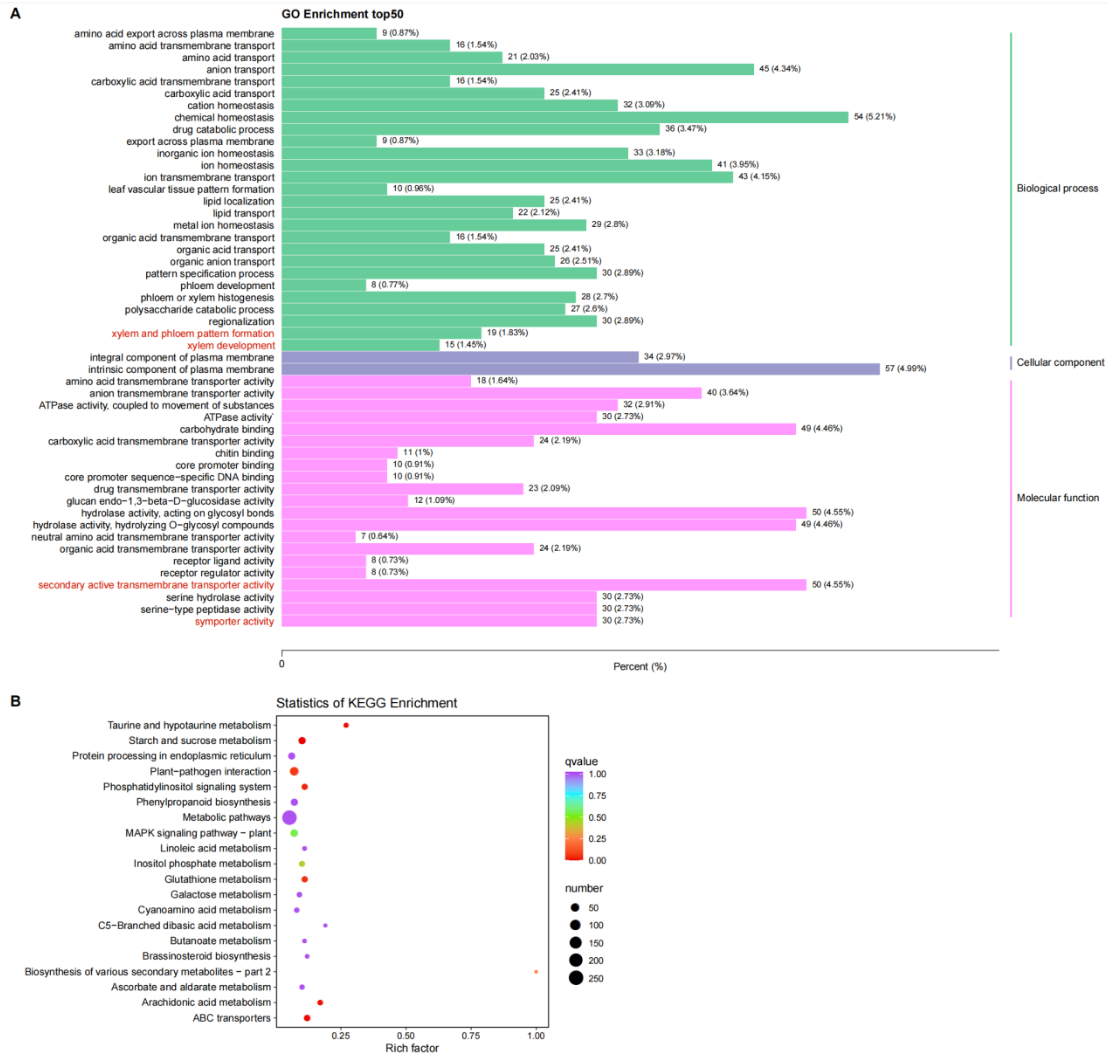

Fig. S6. Functional Annotation of differentially Expressed Genes Between Locule and Placenta Tissues of All-Flesh Fruit Tomato. (**A**) The significantly enriched GO terms; (**B**) The significantly enriched KEGG pathways.

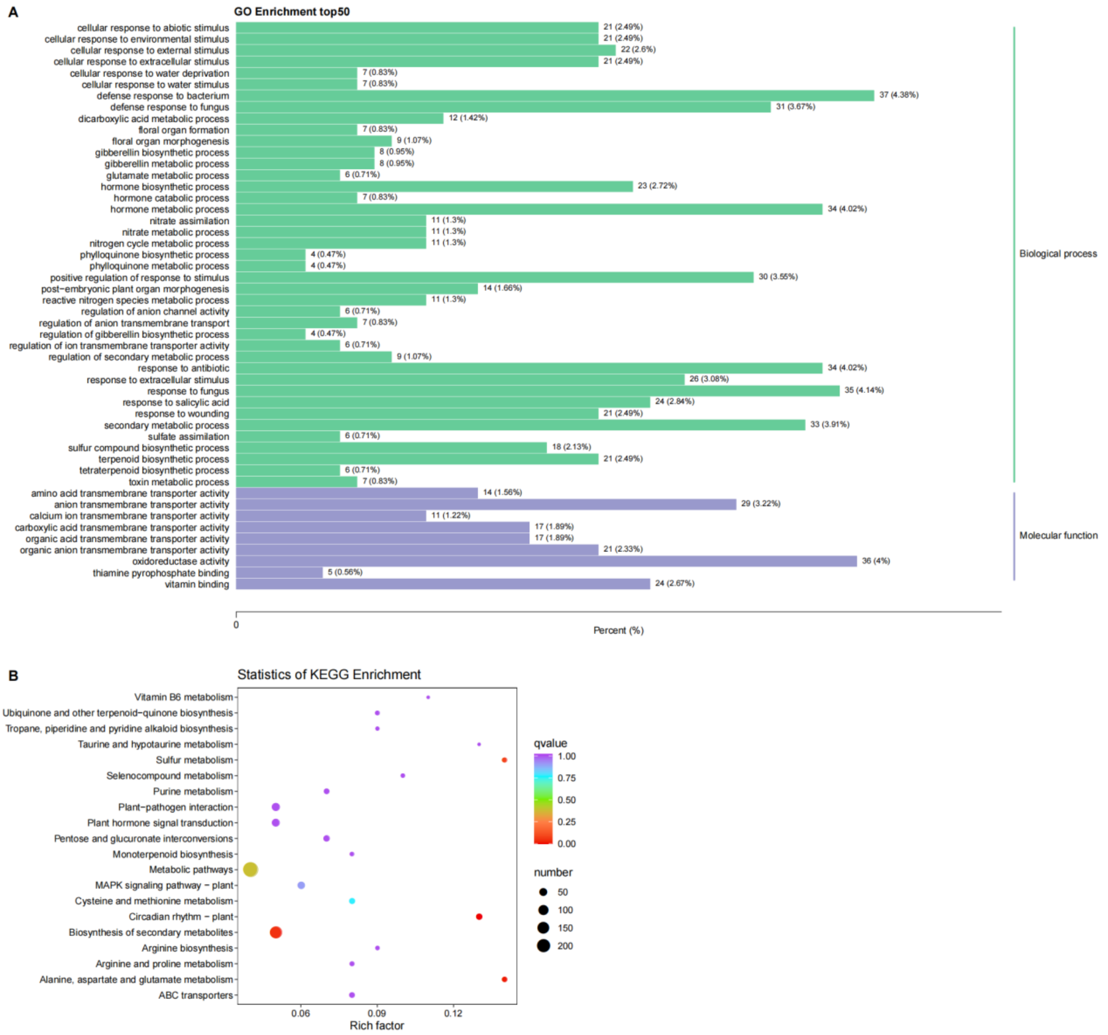

Fig. S7. Functional Annotation of Differentially Expressed Genes in Placenta Tissues Between the Wild-Type and All-Flesh Fruit Tomato. (**A**) The significantly enriched GO terms; (**B**) The significantly enriched KEGG pathways.

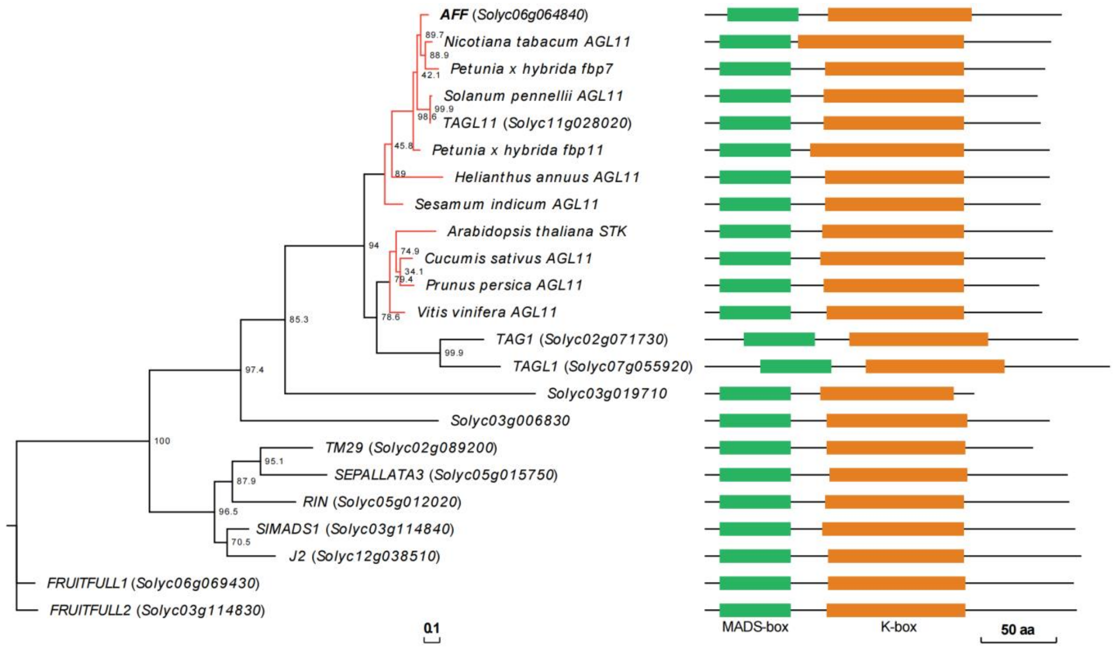

Fig. S8. **Phylogenetic Tree of AFF and Homologous AGAMOUS Proteins in Tomato and Other Closely Related Species.** The phylogenetic clade of the D-class MADS-box proteins were highlighted by the red branches. The right panel indicates the functional domains in corresponding proteins.
